# Practical Notes and Formulæ

**Published:** 1884-11-20

**Authors:** 


### PRACTICAL NOTES AND FORMULÆ.

**Treatment of Acute Rheumatism.**—Dr. J. W. Futrell writes to the Louisville Medical News on the treatment of acute inflammatory articular rheumatism. He says :

I have tried many remedies, but none have yielded me the uniform satisfaction I have derived from the following agents, simple as they may seem to be—so simple, indeed, and so very old-fashioned, that some of your readers may not be willing to prescribe them. I hope, however, that none will be deterred on account of the reasons named, and that when called on to treat their next case of rheumatism they will give the drugs named an impartial trial.

I begin the treatment by putting one ounce each of Epsom salts, nitrate of potash, and powdered sulphur into one quart of boiling water. This, after being allowed to stand in a covered vessel for six hours, is well strained and given in doses of one ounce three or four times during the day.

To the swollen and painful joints I apply, by means of cloths, a liniment composed of—

℞ Olive oil........................................℥ v.

Chloroform ....................................℥ ij.

Hartshorn.......................................ʒ vj.

Tincture of aconite root........................ʒ ij.

M. Apply sufficiently often to relieve pain.

Should the above means not secure rest at night, I administer a full dose of bromide of potash, and repeat in one hour, if necessary.

I have now used it in quite a number of cases of acute and even violent rheumatism, and have not seen it fail to do good.—*Drug Cir.*

**Upham's Asthma Remedy.**—

℞ Pulv. stramonium leaves.........................℥ viij

Pulv. skunk cabbage..............................℥ viij

Pulv. lobelia.....................................ʒ vj.

Mix. Dissolve four ounces of nitrate of potassa in one pint of water, and mix well with the powder. Dry thoroughly and smoke in an ordinary clay pipe morning and evening.—*Ib.*

**Cholera.**—Dr. Burdger, in the Medical World, recommends the following

℞ Magn. carb.......................................ʒ j

Sacch. alba.......................................ʒ ij

Tinct. nucis. vom................................fʒ j

Æther sulph. }

Aq. anisi.... } aa...............................f℥ ij

M. Sig : Agitate the contents of the vial and administer one tablespoonful every half hour. This is a dose for an adult. Keep the vial tightly corked.

**Quinine.**—Of the many devices to correct the bitterness of quinine the following are selected :

No. 1.

℞ Fluid extract yerba santa........................parts 4
Water........................................" 8
Powdered pumice.................................." 1
Granulated sugar................................." 14

Mix the fluid extracts with the water ; evaporate to 7 parts ; shake with pumice ; allow to stand ; decant ; add sufficient water to preserve measure, then with heat dissolve the sugar. The addition of fluid extract of licorice, in the proportion ef one half drachm to the ounce of sirup, or of aromatics, adds somewhat to the elegance of the preparation. Suspend the quinine in the preparation.

No. 2.

℞ Tr. orange peel (recent).......................fl. ℥ 2

Ext. licorice (fluid)..............................fl. ℥ 6

Sirup.............................................fl. ℥ 1

Mix.

No. 3.

℞ Coffee.............................................℔ 1

Chocolate..........................................℥ 8

Glycerine..........................................℥ 40

Sugar..............................................℥ 16

Extract of vanilla.................................℥ 2

Alcohol............................................℥ 13

Water q. s.

Melt the chocolate in the glycerine, and add to it the sugar previously dissolved in 4 pints of water ; make an infusion of the coffee, so as to obtain 2 pints of liquor, and mix this with the first solution ; lastly, add the vanilla and alcohol.

Compound elixir of taraxicum or sirup of licuorice are sometimes used. The bitterness of most drugs can be removed from the mouth by chewing licorice root or tea leaves just before and after the medicine is taken.—*Nat. Druggist.*

**Squibb's Diarrhœa Mixture.**—

℞ Tr. opii........ }

Spts. camphor... } aa..............................℥ j

Tr. capsici...... }

Chloroformi purif..................................ʒ iij

Alcoholis fort.....................................℥ v

M. Dose for adult, one teaspoonful.

In time of epidemic cholera, when a person has two movements of the bowels more than natural within twenty-four hours, the second should be followed by a dose of this mixture, to be repeated after every movement that follows. Immediately after the first dose, the person should go to bed and remain there for twelve hours after the diarrhœa has entirely ceased.—*Med. World.*

**Dysentery.**—A writer in the Medical World says: The best treatment for dysentery I have ever tried is as follows:

℞ Potass. bitart.......................................℥ ij
Pulv. ipecac.......................................gr. ij
Pulv. rhei.......................................gr. iv.

M. et ft. chart. No. iv. Sig: One every three hours until stools pass like water without tenesmus.

After this I give:

℞ Pulv. ipecac. comp.......................................gr. xx
Quinia sulph.......................................gr. viij
Sodæ bicarb.......................................gr. xv.

M. et ft. chart. No. viij. Sig: One every four hours until stools become natural.

REMARKS.—Should tenesmus appear in a short time, give first prescription again.

If mucous discharges (without blood) persist, give:

℞ Ammon. mur.......................................ʒ ss

Pulv. ipecac.......................................gr. x

M. et ft. chart. x. Sig: One every four or five hours, in mucilage.

A parasiticide that has given us good satisfaction is powdered borax ʒ ss, salicylic acid ʒ iii; rub these in a mortar till they become pasty, then add glycerine ℥ ij; rub well, and add water ℥ ii. This has cured pimples, eczema, itch, and other skin diseases of a parasitic nature. All other parasiticles have proven objectionable in our experience, and, by the way, the above is a splendid application in erysipelas as well as a good anticeptic in surgery.—*Georgia Ec. Med. Jour.*

**To remove Rust Stains from Cotton and Linen.**—A mixture of two parts powdered cream tartar with one part powdered oxalic acid is said to give better results than when only oxalic acid is used. Apply little of the powder to the dampened goods.—*Ib.*

**Government Harness Dressing.**—Mix well together with a gentle heat:

Neatsfoot oil.......................................1 gal.

Bayberry tallow.......................................2 lbs.

Beeswax.......................................2 "

Beef tallow.......................................2 "

Castor oil.......................................2 qts.

Lampblack.......................................1 oz.

Strain while hot through fine cloth, and allow to cool. Said to be a superior dressing.—*Nat. Laundry Jour.*

